# International humanitarian narratives of disasters, crises, and Indigeneity

**DOI:** 10.1111/disa.12576

**Published:** 2023-04-26

**Authors:** Anuszka Mosurska, Aaron Clark‐Ginsberg, James Ford, Susannah M. Sallu, Katy Davis

**Affiliations:** ^1^ PhD Researcher at the Priestley International Centre for Climate University of Leeds United Kingdom; ^2^ Behavioral and Social Scientist at the RAND Corporation United States; ^3^ Priestley Chair in Climate Adaptation at the Priestley International Centre for Climate University of Leeds United Kingdom; ^4^ Associate Professor at the Sustainability Research Institute, School of Earth and Environment University of Leeds United Kingdom; ^5^ PhD Researcher at the Priestley International Centre for Climate University of Leeds United Kingdom

**Keywords:** communication, emergency management, disasters, humanitarian action, Indigenous Peoples, narratives

## Abstract

Narratives are a means of making sense of disasters and crises. The humanitarian sector communicates stories widely, encompassing representations of peoples and events. Such communications have been critiqued for misrepresenting and/or silencing the root causes of disasters and crises, depoliticising them. What has not been researched is how such communications represent disasters and crises in Indigenous settings. This is important because processes such as colonisation are often at the origin but are typically masked in communications. A narrative analysis of humanitarian communications is employed here to identify and characterise narratives in humanitarian communications involving Indigenous Peoples. Narratives differ based upon how the humanitarians who produce them think that disasters and crises should be governed. The paper concludes that humanitarian communications reflect more about the relationship between the international humanitarian community and its audience than reality, and underlines that narratives mask global processes that link audiences of humanitarian communications with Indigenous Peoples.

## نبذة مختصرة

الروايات هي وسيلة لفهم الكوارث والأزمات. ينقل القطاع الإنساني القصص على نطاق واسع، ويحمل معها تمثيلات الشعوب والأحداث. تم انتقاد مثل هذه الاتصالات لتحريفها و / أو إسكات الأسباب الجذرية للكوارث والأزمات، وعدم تسييسها. ما لم يتم بحثه هو كيف تمثل هذه الاتصالات الكوارث والأزمات في أماكن السكان الأصليين. وهذا الأمر مهم لأن عمليات مثل الاستعمار غالبًا ما تكون السبب الجذري لكارثة الشعوب الأصلية، ولكنها عادةً ما تكون مُقنَّعة في الاتصالات. نحدد ونوصف السرد في الاتصالات الإنسانية التي تشمل الشعوب الأصلية من خلال إجراء تحليل سردي للاتصالات الإنسانية. نحدد خمس روايات: تصرُّف العاملون في المجال الإنساني، وإسناد الذنب، والناس يساعدون الناس، والأمة تتصدى للكوارث، ونبتكر طريقنا للخروج من الكارثة. تختلف الروايات بناءً على الكيفية التي يعتقد بها العاملون في المجال الإنساني الذين ينتجونها أن الكوارث والأزمات يجب أن تُحكم. خصصت معظم المقالات مساحة للعمل الإنساني، بينما ركزت مقالات أخرى على مشاهدة الجماهير الدولية وإعداد التقارير عنها وتحمل مسؤوليتها. نستنتج أن الاتصالات الإنسانية تعكس العلاقة بين المجتمع الإنساني الدولي وجمهوره أكثر مما تعكس الواقع، وتفكر في كيفية إخفاء الروايات للعمليات العالمية التي تربط جمهور الاتصالات الإنسانية بالشعوب الأصلية.

**الكلمات المفتاحية **: العمل الإنساني، الكوارث، الروايات، الشعوب الأصلية، الاتصالات، إدارة الطوارئ

## 摘要

记叙是理解灾难和危机的一种方式。人道主义部门广泛传播故事，讲述人物和事件。这些传播被批评为歪曲和/或压制灾害和危机的根源，使其非政治化。尚未研究的是此类传播如何在原住环境下代表灾难和危机。这点很重要，因为殖民等过程通常是原住居民灾难的根本原因，但在传播过程中常常被掩盖。我们通过开展人道主义传播的记叙分析来识别并特色化描述涉及原住居民的人道主义叙事。我们确定了五种叙述方式：人道主义者行动、追究责任、人民帮助人民、国家应对灾难以及创新摆脱灾难的方式。记叙方式的不同取决于叙事的人道主义工作者认为应该如何治理灾难和危机。大多数文章为人道主义行动开辟了空间，而其它文章则侧重于见证、报道，赋予国际读者责任感。我们得出结论，人道主义传播更多地反映了国际人道主义界及其受众而非现实之间的关系，并反思了叙事方式是如何掩盖这一将人道主义传播的受众与原住居民联系起来的全球进程。

**关键词**:人道主义行动、灾害、叙事、原住居民、传播、应急管理

## Introduction

The stories we tell are ways of making sense of the world, especially when disasters and crises happen. Language serves as a legitimiser of events and ideas, so narrative analysis is an appropriate method to reveal identities, shared (and not shared) values about society, and exploratory reasoning as imagined by humanitarian actors (Vincent, 2000; Barnett, 2013). This is because narratives are a means of understanding the social world (Barkin and Gurevitch, 1987; Somers, 1994), so our ability to interpret it increases as we master the various narratives and begin to employ them (Vincent, 2000).

A narrative can be defined as an account of a series of actions and events, unfolding over time, in which characters encounter trouble and strive to resolve or survive it (Bruner, 2004). Characters act in ways that are meaningful (that is, they have social significance), and actions are undertaken with motive (the emotionally charged desire to achieve or prevent something) (Mroz, Papoutsi, and Greenhalgh, 2021).

When looking at how others utilise narratives, we can comprehend how agents represent, legitimate, and contest order (Spandler, 2022). As narratives provide knowledge about ‘how things should be’, by examining shared understandings in narratives, we reveal the values of the society in which humanitarian actors live (Wong and Breheny, 2018).

When powerful and visible actors tell stories, their interpretations, meanings, and values are communicated to vast audiences. In the humanitarian and disaster response sector, narratives, and the representations involved, are one of the principal tools used to leverage power in the form of money, public outcry, governmental attention, or some other social process or outcome (Norton, 2011). Therefore, narratives have a clear impact on practice.

Combined with this is an increasingly competitive atmosphere among aid agencies, which need to compete for visibility and donors (Chouliaraki, 2013). As a result, the humanitarian media and communications sector is rapidly growing, with a focus on the role of narrative and images in provoking moral responses, cultivating care, compassion, responsibility for, and action aimed at alleviating the suffering of distant strangers (Orgad and Seu, 2014; Wasif, 2020).

We identify, characterise, and scrutinise here humanitarian actors' narratives of humanitarian action during disasters and crises involving Indigenous Peoples. We adopt a narrow and normative definition of ‘humanitarians’, following Fiddian‐Qasmiyeh's (2019) definition of the ‘international humanitarian community’, which is hegemonic and composed, inter alia, of United Nations (UN) agencies and international non‐governmental organisations (INGOs). We recognise that this is as one of a plurality of international communities of response to disasters and crises, but spotlight this community as it is so visible and powerful in setting the discourse and humanitarian agenda.

We focus on Indigenous Peoples for three reasons. First, the localisation and decentralisation of humanitarian action, disaster risk reduction (DRR), and aid has been at the top of international policy agendas, such as the World Humanitarian Summit and the Intergovernmental Panel on Climate Change (IPCC) (Al‐Abdeh and Patel, 2019; Hendriks and Boersma, 2019; Gómez, 2021). As a part of this, Indigenous Peoples have become a key target of and actor in humanitarian initiatives (Ali et al., 2021). Yet, humanitarian initiatives and disaster management can be colonising in Indigenous settings because they change Indigenous communities based on outsider values and/or maintain the status quo in line with colonial agendas (Saini, 2018). In response, there have been drives to ensure such action is culturally appropriate (Yumagulova et al., 2020). Little is known about whether these changes are reflected in humanitarian communications.

Second, the case of Indigenous Peoples matters because they exist in spaces where the legitimacy of government is contested in ways that are different to other marginalised groups (Siddiqi and Canuday, 2018). Unlike many marginalised groups that seek to achieve equality within a nation‐state, Indigenous Peoples are frequently working towards a degree of independence from nation‐states (Arvin, Tuck, and Morrill, 2013). Throughout this paper we pay particular attention to issues of governance, keeping in mind specifically the self‐determination of Indigenous Peoples.

Third, non‐Indigenous audiences are often unfamiliar with Indigenous politics (such as self‐determination) (see, for example, Merino, 2020). This amplifies the importance of humanitarian communications as a source of information. In these contexts, such organisations are powerful in representing disasters and crises in Indigenous settings, yet they have not been previously analysed. In our discussion we argue that these narratives mediate the relationship between audiences and those affected by such events in ways that mask how audiences may be complicit in unequal power relationships. Drawing on care ethics—a relational and feminist approach to care—we assert that audiences do not view themselves as helping ‘distant strangers’, but rather as embedded within unequal power relations that place different groups of people at risk. In doing so, inequalities across global scales are brought to the fore.

## The context: disasters, humanitarian response, and Indigenous Peoples

### Disaster and humanitarian narratives

What constitutes a disaster, crisis, or emergency is contested, as these constructs are not objective but categories that signify a problem defined by someone, usually an organisation with some form of power (Anderson et al., 2020; Bandopadhyay, 2022). There has been a steady reframing of disasters, moving from abnormal, natural, unpredictable events, to socially constructed processes that build up over time (Hewitt, 1983), often aligned with concepts such as slow violence—mundane, creeping, and regularly ignored violent processes—and necropolitics—the subjugation of life to the power of death (Mbembé and Meintjes, 2003; Nixon, 2009). These concepts recognise that unequal power structures place some people at greater risk in ways that are frequently invisible. In this way power inequalities are recognised as root causes of disasters, rather than the environment.

Once an emergency is declared, who should act and what should be done is shaped through labels, categorisations, narratives, and other discursive constructions. For instance, how human suffering is portrayed calls on humanitarians to be present on the ground with their staff, values, and toolkits, carrying the assumption that humanitarians and their toolkits are relevant, useful, and welcome (Dijkzeul and Sandvik, 2019). Narratives about Indigenous Peoples often misrepresent them through essentialisation, sometimes constructing them as deviant to justify intervention (de Leeuw, Greenwood, and Cameron, 2010; Tsai et al., 2020). These categories signal the creation of fictionalised enemies, objects/subjects in danger, agents ideally placed to undertake rescue, and social and political needs (Mbembe, 2008; Dijkzeul and Sandvik, 2019; Khoja‐Moolji, 2020). Thus, humanitarianism has emerged as a global discourse that relies and reproduces unequal geopolitical relationships through catastrophic images (and text) about the ‘other’, who are constantly suffering (Tascón, 2017).

Narrativising—the process of depicting a setting, characters, and a meaningful sequence of events and actions unfolding over time—helps people deal with disasters, such as through understanding them and galvanising collective action (Bendix, 1990; Chamlee‐Wright, 2018; Mroz, Papoutsi, and Greenhalgh, 2021). Yet, narratives about disasters may constrain understanding of such events and those involved in them. Narratives are never neutral but told from certain perspectives and social locations. They are intertwined with processes of meaning‐making that reinforce particular social, political, and theological frameworks (Belser, 2015). For example, in both disaster and humanitarian research, harm is linked to broad structural issues and power inequalities that unevenly distribute risk, such as oppression (Lewis, 1976; Mbembé and Meintjes, 2003; Ong, 2019), although many humanitarian actors use framings of disasters as unpredictable to justify short‐lived emergency relief efforts that do not consider the political economy of places (Fiddian‐Qasmiyeh, 2019). With this, the role of certain actors (such as the State) in vulnerability creation is obscured (Carrigan, 2010). In addition, activities viewed as deeply political are reduced to matters of techniques and bureaucracy, and left to ‘experts’ (Ferguson, 1990; Howitt, Havnen, and Veland, 2012).

### The humanitarian sector

We centre our analysis on the most visible and powerful part of the humanitarian sector: the normative, Northern‐led ‘international humanitarian community’ (Fiddian‐Qasmiyeh, 2019). Actors within this community have been criticised for driving the corporatisation of humanitarianism (Chouliaraki, 2010, 2013), while their collaboration with local nongovernmental organisations (NGOs) has been critiqued as symbolic, bolstering the legitimacy of the international humanitarian community without political or economic commitment (Wright, 2018). This group of actors has also been criticised for structural racism, including a monopoly on and misuse and abuse of power, alongside resistance to decolonisation (Aloudat and Khan, 2022; Majumdar and Mukerjee, 2022). However, the humanitarian sector is incredibly diverse (Fiddian‐Qasmiyeh, 2019). Some choose to work within existing power structures (such as with national governments), whereas others work outside of them (such as in advocacy) (Stoddard, 2003; Akbarzadeh, Barlow, and Nasirpour, 2021). Religious actors often seek to bridge secular and religious worlds by concentrating on social issues (Wilkinson, 2018), whereas others are more operations‐focused (Stoddard, 2003). There is also increased regionalisation of humanitarian assistance (such as South–South collaboration) (Fernando and Hilhorst, 2006; Ong, 2019), recognition of informal and local disaster response (Lewis, 2019; Duda, Kelman, and Glick, 2020), North–South collaboration (Charles, Lauras, and Tomasini, 2010; Vukojević, 2013), and responsibilisation of the private sector (Atal and Richey, 2021). These shifts in governance (Tierney, 2012) are complemented by paradigm shifts within humanitarianism, such as moves away from traditional humanitarianism (focused on immediate response and addressing basic needs) towards ‘resilience’ humanitarianism, which seeks to address underlying, structural causes of harm (Hilhorst, 2018). Despite the rise in this approach, enacting the type of change required to resolve root causes of vulnerability is challenging. Generally, humanitarian action addresses the capacities of people in the face of structural vulnerabilities, which may or may not also be tackled (see, for example, Abdelnour and Saeed, 2014).

### Narratives in humanitarian communications

Humanitarian communications do not necessarily reflect the practices of humanitarians. Instead, they are constructed by humanitarians based on ways they believe will bolster their credibility in the eyes of audiences (Gourevitch and Lake, 2011). This matters because, through the use of carefully constructed narratives, humanitarian communications call upon audiences to care for and act in solidarity with distant others (Abraham, 2015; Gill, 2020), while positioning humanitarians in relation to events in ways that justify their intervention on moral grounds (Givoni, 2011). They also may present corporations and consumers as actors who can solve problems, distracting from inequality, and repositioning them as heroes, rather than agents implicated in unequal power relations (Richey, 2018).

Humanitarian communications have been critiqued for depoliticising disasters, often by masking the root causes of events and structural inequalities by focusing on suffering and basic needs (Gill, 2020). Thus, humanitarian communications have been criticised for inhibiting complex debates about the root causes of disasters, and radical solutions to them, which include addressing neoliberalism and forms of North–South dependence (Lugo‐Ocando, 2014). Such simplification and misrepresentation of disasters is reflective of the market logics imbued in humanitarian communications: emotions of potential donors are a scarce resource for which humanitarians compete (Gill, 2020). As a practice that is under constant scrutiny (Orgad, 2017), and which is used to leverage power and funds, it is necessary to analyse and critique carefully humanitarian communications, as well as to think about other ways of communicating. For instance, in an effort to bring power relations to the fore, Gill (2020) and Houbeish (2021) argue for care ethics in humanitarian communications, where mutual concern and trust is built. They contend that doing so challenges colonial stereotypes, lessening the viewing of people as distant sufferers and rather as people connected to each other through complex power relations. This is important because it refocuses attention on the causes of disasters and crises, rather than concentrating solely on suffering.

### Indigenous Peoples in disasters and humanitarian response

The United Nations Declaration on the Rights of Indigenous Peoples (UNDRIP), adopted in 2007, states that ‘Indigenous’ should be understood in reference to a community of peoples sharing intergenerational ancestry and cultural aspects with original (pre‐colonial) occupants of ancestral lands in a specific region of the world (United Nations, 2007). Given their different histories and diversity, there are some fundamental differences between Indigenous worldviews and non‐Indigenous ones that can shape narratives. We outline some of these as they pertain to the fields of disasters and humanitarianism. Fundamentally, communitarianism, collectivism, and relationality are critical to many Indigenous groups (Chávez Ixcaquic, 2014; Banerjee, 2016; Karides, 2016). These relations extend beyond humans, as non‐humans and more‐than‐humans are viewed as sentient (Lozano, 2016; Yazzie and Baldy, 2018; Richardson–Ngwenya, 2021). Drawing on the work of Lorena Cabnal (2015)—an Indigenous communitarian Maya‐Xinka feminist—on *Cuerpo‐territorio* (body‐territory), community and territory are viewed as a single subject of political agency that resists and identifies violations against women's bodies and territories as a part of the same process (Mollett, 2021). In many contexts, Indigenous women organise against neoliberal processes, particularly extractivism (Kuokkanen, 2019; Santamaria et al., 2019), and play an important role in Indigenous communities, indicating how many Indigenous groups had very different theories on gender before colonisation, and that heteropatriarchy was imported through colonialism (Kim, 2020). In Chuukeee ontology, for example, all beings are connected through maternal creation. Women embody the environment, making visible in human domains its broader role in giving birth to all living things, human and non‐human, spirits and non‐spirits. With this, then, many Indigenous Peoples have different views of time and death, with inter‐generational responsibilities extending to descendants and ancestors (Ratuva, 2007; Matiure, 2011).

When humanitarians, especially powerful members of the international humanitarian community, are unfamiliar with Indigenous worldviews, they may fundamentally change Indigenous communities based on outsider values (Saini, 2018). Regularly, such engagements are frustrated by positivist, technical, and managerial approaches to disasters that do not recognise the role of settler colonialism in the production of risk and vulnerability (Thomassin, Neale, and Weir, 2019). Thus, humanitarian action has been considered a form of colonialism in some Indigenous contexts (Watson, 2017). Ongoing impacts of colonialism, such as this, affects Indigenous Peoples' ability to deal with disasters, frequently eroding Indigenous institutions (Saini, 2018). The State often fails to take Indigenous expertise seriously (Thomassin, Neale, and Weir, 2019) and there is discrimination against Indigenous Peoples in many contexts (Wambrauw, 2017). Stereotypes can filter into disaster management, so there needs to be space for Indigenous Peoples to bring forward their own worldviews and knowledge (Lyons et al., 2020). Aside from guarding against harmful stereotypes and ensuring that Indigenous Knowledge and culturally appropriate Indigenous institutions are drawn upon in disaster management (Cuaton and Su, 2020; Yumagulova et al., 2021; Quinn, Williamson, and Gibbs, 2022), doing so is a step towards self‐determination of Indigenous Peoples: a foundational right and principle that ensures Indigenous Peoples choose how they are governed (Napoleon, 2005).

## Methodology

To identify articles, we searched four websites: PreventionWeb (similar to the study by Chmutina, von Meding, and Bosher (2019) on language in DRR); ReliefWeb; United Nations Development Programme (UNDP); and the UN Permanent Forum on Indigenous Issues. We chose this approach as these sites included communications of many INGOs and agencies that are reflective of the international humanitarian community. It facilitates interrogation of the most powerful and visible organisations.

Although the term ‘Indigenous’ is widely used and accepted by the UN, it is not universally accepted, with some groups preferring to use nouns such as ‘tribes’ (Banerjee, 2016). Therefore, while ‘Indigenous’ produced the most results, we also searched for synonyms such as ‘tribe’. This was an iterative process that involved conducting test searches to determine how to generate the highest number of relevant articles. We performed searches in all six official UN languages. Inclusion criteria were that the author organisation had to be discussing its own initiatives in the context of a disaster and included at least one paragraph that focused on Indigenous Peoples. Following Ahmad (2018), we view the disaster–non‐disaster binary as unhelpful. As such, we move away from seeing disasters as discrete events and include processes such as climate change, structural violence, and conflict in our inclusion criteria. We embrace the messiness of such an approach and consider the disaster/problem identified in the article an element of our analysis.

There is no definitive approach to conducting a narrative analysis, and researchers have done so heterogeneously and flexibly to fit their needs (Polkinghorne, 1995; Wong and Breheny, 2018). Narrative researchers interpret meaning through analysis of plot‐lines, thematic structures, and social and cultural referents (Kim, 2016). Given our aim to identify themes that are common, and those that diverge, across different texts as well as in relation to patterns, narrative threads, tensions, and themes (Kim, 2016), we follow Polkinghorne's (1995) paradigmatic analysis of narrative data, rather than narrative analysis per se (which seeks to consolidate various narratives into one, but not necessarily to analyse them further). Following Labov (1972), the following were coded deductively:

- Abstract (the summary of the story).
- Orientation (when and where the events occurred and which characters were involved. We included more‐than‐humans and non‐humans as actors if they were framed that way, since many Indigenous ontologies view elements of the environment as sentient).
- Complicating action (sequences of action that move the plot forward); evaluation (narrator's comments and interpretations of the story); and resolution (the outcome of the story).
- Coda (the ending clauses of the narrative).

We combined this deductive approach with grounded thematic coding (Chien, 2019) through first cycle coding methods (such as initial coding) (Saldaña, 2013). Next, we grouped codes into categories and eventually themes. Overall, we retained 95 articles for analysis, of which 94 were in English and one was in Spanish.

## Results

Of the 95 articles, most focused on South and Central America, Southeast Asia, and Sub‐Saharan Africa, although a minority also included Small Island Developing States (SIDS), the Middle East, North Africa, and North America.

**Figure 1:**
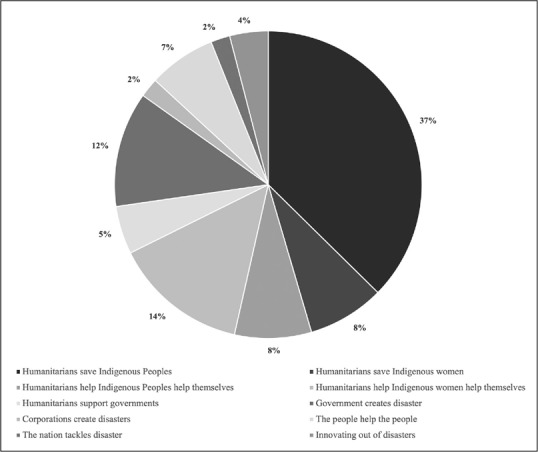
Percentage of each narrative in the sample **Source**: authors.

We identified five main narratives: humanitarians act (78 per cent of articles); the nation tackles disaster (2 per cent of articles); the people help the people (7 per cent of articles); attributing culpability (14 per cent of articles); and innovating out of disasters (4 per cent of articles). These were exclusive (that is, each article aligned with one narrative only). We further identified five sub‐narratives in the ‘humanitarians act’ narrative: humanitarians save Indigenous Peoples (37 per cent of articles); humanitarians save Indigenous women (8 per cent of articles); humanitarians help Indigenous Peoples help themselves (8 per cent of articles); humanitarians help Indigenous women help themselves (14 per cent of articles); and humanitarians support governments (5 per cent of articles). We also identified two sub‐narratives within the attributing culpability narrative: government creates disaster (12 per cent of articles); and corporations create disasters (2 per cent of articles) (see Figure 1).

Table 1 contains a breakdown of narratives by organisation, country, and disaster.

**Table 1:** Narratives identified and the organisations, countries/regions of focus, and disasters involved

Narrative	Sub‐narrative	Description	Organisations	Country/region	Disasters
Humanitarians act	Humanitarians save Indigenous Peoples	Set amidst crisis, humanitarians act to rescue Indigenous Peoples from disaster, mostly focusing on basic needs.	Asian NGOs European NGOs Intergovernmental agencies International faith‐based NGOs International NGOs	Central America Middle East North Africa South America South Asia Southeast Asia Sub‐Saharan Africa	Armed conflict^3^ Environmental disasters (including climate change) Pandemics and epidemics Political disasters (such as statelessness) Unsafe living conditions (such as lack of clean water)
	Humanitarians save Indigenous women	Set amidst crisis, humanitarians act to rescue Indigenous women from disaster, mostly focusing on gendered aspects of disasters.	Intergovernmental agencies International faith‐based NGOs International NGOs	Central America North Africa Southeast Asia	Armed conflict Environmental disasters (such as flooding) Pandemics and epidemics Unsafe living conditions (such as childbirth risks) Violence (such as gender‐based violence)
	Humanitarians help Indigenous Peoples help themselves	Set amidst crisis, humanitarians act to empower Indigenous Peoples to help themselves.	Intergovernmental agencies International faith‐based NGOs International NGOs	Central America SIDS South America South Asia Sub‐Saharan Africa	Armed conflict Environmental disasters (such as typhoons) Pandemics and epidemics Political disasters (such as illegal mining)
	Humanitarians help Indigenous women help themselves	Set amidst crisis, humanitarians act to empower Indigenous women to help themselves. Often focused on the unique characteristics of Indigenous women that places them as ideal recipients of aid and agents of change.	Climate‐focused international NGOs Intergovernmental agencies International faith‐based NGOs	Central America SIDS South America Sub‐Saharan Africa	Environmental disasters (including climate change) Pandemics and epidemics Unsafe living conditions (such as female genital mutilation, hunger) Violence (such as gender‐based violence)
	Humanitarians support governments	Set amidst crisis, humanitarians support the (non‐Indigenous) government, which then supports Indigenous Peoples.	Intergovernmental agencies	Central America SIDS South America	Armed conflict Environmental disasters (including climate change)
Attributing culpability	Government creates disaster	Nation‐states create a crisis and/or make an existing crisis worse. They break international law and so humanitarians draw attention to this.	International advocacy groups International faith‐based NGOs Intergovernmental agencies	North Africa South America South Asia Southeast Asia Sub‐Saharan Africa	Armed conflict Environmental disasters Pandemics and epidemics (such as COVID‐19) Political disasters (such as police raid on schools) Unsafe living conditions (such as hunger)
	Corporations create disasters	Corporations create a crisis and/or make an existing crisis worse, sometimes with the help of national governments. They break international law and so humanitarians draw attention to this.	Intergovernmental agencies	Central America South America	Pandemics and epidemics Political disasters (such as mining)
The people help the people	–	There is a crisis where the global community is ignoring Indigenous Peoples. The only people who will help are those on the ground. Grassroots action is needed.	Intergovernmental agencies International NGOs	Central America Middle East SIDS South America South Asia	Environmental disasters (such as a hurricane) Pandemics and epidemics (such as AIDS (acquired immune deficiency syndrome)) Unsafe living conditions (such as a lack of rights)
The nation tackles disaster	–	There is a crisis but the government steps in. Owing to government action, the crisis is over.	Intergovernmental agencies	North Africa South America	Pandemics and epidemics (such as COVID‐19) Unsafe living conditions (such as discrimination)
Innovating out of disasters	–	Disasters are on the rise. By combining Indigenous knowledge with Western science, technological innovation prevents disasters.	Climate‐focused NGOs Intergovernmental agencies	North America SIDS South America	Environmental disasters (including climate change)

**Source**: authors.

### Humanitarians act

‘Humanitarians act’ was the most dominant narrative and comprised articles that primarily focused on the ways that humanitarian actors assisted Indigenous Peoples. It included five separate sub‐narratives, which we outline below.

#### Humanitarians save Indigenous Peoples

In this sub‐narrative, Indigenous People suffer (especially Indigenous women and children) as their basic needs are not met. As colonised people (EU (European Union), 23 September 2020),^2^ they live in harsh environments, and are forgotten about. Indigenous Peoples are vulnerable and do not have the capacity to deal with a disaster. They pay tribute to Mother Earth (Benrey, 16 August 2021), but the disaster has killed the environment upon which they rely (EU, 2 December 2020). In some instances, God is angry at the pollution of Indigenous lands by non‐Indigenous Peoples (Macheka, 16 October 2021). God has sent warnings in the form of drought and has encouraged Indigenous Peoples to move to the mountains. The mountains, where humanitarian projects are cleaning the water, protect Indigenous Peoples (Macheka, 16 October 2021). Humanitarians work hard (MSF (Médecins Sans Frontières), 17 June 2020) to respond and stabilise the situation. They teach Indigenous Peoples and bring ‘the light of education’ (Jahan, 19 May 2021). Indigenous Peoples are happy and thankful for humanitarian action, and there is a strong community spirit around humanitarian action (Act Alliance, 28 February 2019; CFSI (Community and Family Services International), 2 February 2021), with many viewing aid as a gift (WFP (World Food Programme), 16 August 2021). However, more is needed, so humanitarian projects should be expanded (Caritas, 16 August 2019; Salesians, 2 March 2021).

#### Humanitarians save Indigenous women

As a subset of the humanitarians act narrative, this sub‐narrative depicts Indigenous women in crisis: there is gender inequality, Indigenous women suffer, are vulnerable, are burdened, and work in the precarious domestic sector (EU, 2 December 2020). The environment, which keeps people alive, is polluted (Tadesse, 19 April 2021). Tribal disagreements make matters worse (ICRC (International Committee of the Red Cross), 24 June 2021, 12 August 2021). Indigenous women are unaware of their rights and lack knowledge of topics such as mental health. They are also without social support; humanitarians work to fill this gap. They address Indigenous women's basic needs (ICRC, 14 June 2021) and reduce their exposure to violence by working with Indigenous women and governments (ICRC, 12 August 2021). Indigenous women are grateful for humanitarians. Now, they have more time to look after their children (ICRC, 24 June 2021).

#### Humanitarians help Indigenous Peoples help themselves

In this sub‐narrative, Indigenous Peoples live in a harsh environment with precarious local infrastructure. Although they are in danger, they have Indigenous knowledge and capacities (MSF, 4 August 2020), but do not know how to use these assets. Humanitarians collaborate with local NGOs and Indigenous leaders. Governments and local authorities fail to manage disasters (MSF, 4 August 2020). INGOs finance efforts and, by working together (CDKN (Climate and Development Knowledge Network), 24 August 2020; Sackitey, 10 February 2020), humanitarians teach Indigenous Peoples to unlock their potential. Outcomes include reconciliation and reforms (UNVMC (United Nations Verification Mission in Colombia), 10 June 2021), securing Indigenous land rights, Indigenous stewardship of land (Arozena, 16 April 2018), and protecting Indigenous Knowledge (MSF, 4 August 2020). Eventually, INGOs hand over to local NGOs (MSF, 4 August 2020).

#### Humanitarians help Indigenous women help themselves

Here, Indigenous women are agents of change, but they lack opportunities, especially given the Indigenous women's crisis (UN Women, 31 October 2020; CECI (Centre for International Studies and Cooperation), 6 July 2020). They are trapped in unpaid work due to patriarchal systems and machismo culture (UN Women, 9 August 2016), where men traditionally make decisions. However, Indigenous men are not always effective leaders (UNFPA (United Nations Population Fund), 9 February 2017). Humanitarians catalyse change by working with Indigenous women to challenge discrimination, enabling them to organise and lead disaster management. Indigenous women look out for those most vulnerable, although some do contest their framing as caregivers (UN Women, 23 August 2018). Indigenous women are proud of their efforts, but more needs to be done to include and centre Indigenous women. Humanitarians advocate for women's empowerment, which is also supported by some Indigenous leaders (CECI, 6 July 2020). Solutions include expanded social welfare (CECI, 6 July 2020) and supporting Indigenous‐led businesses (UN Women, 9 August 2016), such as through financial risk management and insurance (WFP, 16 August 2021). Sometimes it is necessary to ban cultural practices, such as female genital mutilation (UNFPA, 9 February 2017).

#### Humanitarians support governments

The fifth sub‐narrative of the humanitarians act narrative portrayed the environment Indigenous Peoples live in as protecting them (del Carmen Sacada, 13 April 2020), and a fundamental part of their culture (UNDP, 16 February 2021). Inequalities exist between Indigenous Peoples and non‐Indigenous Peoples, and Indigenous women particularly suffer as a result. Where they can, Indigenous Peoples exercise their self‐determination, especially when non‐Indigenous Peoples put Indigenous Peoples at risk. Governments are concerned and respond in ways that are culturally appropriate, such as by ensuring communications are in Indigenous languages (del Carmen Sacada, 13 April 2020), allowing for Indigenous burial practices, and including them in community‐based fisheries management (UNDP, 16 February 2021). They work with Indigenous Peoples to design and implement these, and they invite humanitarians to support them in this.

### Attributing culpability

Articles following narratives on culpability attributed fault for creating disasters either to governments or corporations. There was some overlap between them, as sometimes corporate action was encouraged by complicit governments.

#### Government creates disaster

In this case, Indigenous Peoples are disproportionately affected by disasters, many of which the State allows to happen. Governments abuse their power by intimidating and displacing Indigenous Peoples violently, often using the military, police, and paramilitary units to do so. Politically motivated killings feature (Amnesty International, 30 July 2021; Conde, 17 February 2021) and some states target Indigenous children (Conde, 17 February 2021). They destroy the environment upon which Indigenous people rely (Survival International, 11 March 2015). The international community ignores this. Indigenous women in particular are activists and make sacrifices for their community (Pappier, 21 March 2019). Humanitarians stand in solidarity with Indigenous Peoples and raise awareness of their situations. Some governments deny allegations or say that they are working in the interest of Indigenous Peoples, but this is false (Amnesty International, 30 July 2021). Instead, humanitarians show that governments are breaking international law (Amnesty International, 11 March 2021; HRW (Human Rights Watch), 6 March 2021). Humanitarians underline that the international community has a responsibility to challenge discrimination and structural racism, especially where Indigenous Peoples are considered less human (Caux, 11 November 2021) and where there is a global order of profits before people in which all are complicit (MRG (Minority Rights Group), 23 August 2019). This could be achieved through more rigorous legal systems (Amnesty International, 11 March 2021) and protecting the environment, which safeguards Indigenous Peoples (Christian Aid, 25 August 2019). Reform of the humanitarian sector is needed to ensure that humanitarians are not abetting (Survival International, 11 March 2015).

#### Corporations create disasters

In this narrative, the Earth, which usually protects people, is sick (UNDP, 19 March 2021). It can no longer provide a safe home for Indigenous Peoples, who are experiencing rare diseases (Amnesty International, 18 May 2021). Indigenous Peoples and their land are confronting high levels of pollution from industries, which are enabled by governments. Big industries threaten Indigenous Peoples, exploiting them and their resources. For them, disasters, like pollution, are opportunities. Humanitarians document Indigenous Peoples' suffering and call out corporations and the State for their actions and collusion. They put pressure on governments to do something. They show that nature‐based solutions and granting Indigenous land rights would help solve the problem, as Indigenous Peoples look after the land and care about future generations (UNDP, 19 March 2021). Including Indigenous worldviews in REDD+ (efforts to reduce emissions from deforestation and forest degradation, and foster conservation), for example, could be a solution (UNDP, 5 May 2017).

### The people help the people

In this narrative, Indigenous Peoples have long been discriminated against (UNAIDS, 21 December 2015; UN Women, 18 April 2017). Indigenous women face violence (UN Women, 18 April 2017) and Indigenous youth encounter barriers, such as to accessing information on sexual health (UNAIDS, 21 December 2015); ableism is also a problem (UN Women, 18 April 2017, UNDRR (United Nations Office for Disaster Risk Reduction), 26 February 2021). Governments abuse their power, often through the police and paramilitary units (Schiavoni, 20 November 2020). Yet, Indigenous Peoples are activists and Indigenous women in particular are knowledgeable. Some individuals have created change in their communities (GPEI (Global Polio Eradication Initiative), 6 March 2018; UN Women, 18 April 2017). People help each other to shift power and dismantle oppressive systems. These social movements fill the gaps left by government failure. Solutions are those that transfer power and include food sovereignty (Schiavoni, 20 November 2020), human rights (UNAIDS, 21 December 2015), Indigenous feminism (UN Women, 18 April 2017) and intersectionality (UNDRR, 26 February 2021). Humanitarians stand in solidarity with Indigenous Peoples.

### The nation tackles disaster

Here, Indigenous Peoples live in remote areas, which presents a challenge when a disaster strikes. Nevertheless, the nation provides healthcare to those who need it, especially Indigenous women and children. Often Indigenous nurses are enlisted to help with these efforts (WHO (World Health Organization), 19 April 2017). Government is receptive to the concerns of Indigenous women and, where necessary, intervenes with traditions of Indigenous Peoples at the request of Indigenous women (UN Women, 29 April 2018). By working together, the nation overcomes a disaster.

### Innovating out of disasters

In the final narrative, Indigenous Peoples work hard but they live in inadequate buildings. The way the land is currently being used is unsustainable too (CAP (Climate Adaptation Platform), 11 October 2021). Some are considering leaving their homelands as a result (CAP, 11 October 2021). A research centre steps in to redesign homes to meet threats such as climate change (CAP, 30 April 2021). These architectural solutions combine Indigenous knowledge with Western technology, which makes them culturally appropriate and sustainable.

## Discussion

The narratives and sub‐narratives that we identified had certain similarities. Across all narratives, disasters and crises were defined broadly, ranging from natural hazards, to conflict, to political conditions (such as statelessness and exclusion), reflecting the inclusion of slow violence and necropolitics as forms of disaster. This aligns with other calls to focus attention on various forms of violence, especially those that are slow and invisible (Aijazi, 2016; Baird, 2021).

Contrary to previous research, the environment was a critical character in these narratives: sometimes it cared for Indigenous Peoples, sometimes Indigenous Peoples cared for it, sometimes it was the source of disaster, and sometimes it protected people from disaster. Many Indigenous cosmologies perceive the environment and its elements as sentient, and to some degree these narratives nod to these cosmologies. For instance, in Macheka (16 October 2021), Indigenous Peoples in Zimbabwe see droughts as punishment of those who have polluted water, as well as warnings for them to move to the mountains, where there is clean water and humanitarian assistance. However, such a view goes against the belief that disasters are socially constructed, and which frames them instead as ‘acts of God’. Here, there is a complex interweaving of Indigenous cosmologies with disaster paradigms that have long been seen as depoliticised (Goodall, Khalid, and Del Pinto, 2022). We approached this analysis with suspicion as well as faith (Josselson, 2004), and remain wary that Indigenous cosmologies may be co‐opted and used to legitimise humanitarian intervention in ways that depoliticise, especially as those communications that do engage some of these cosmologies do not come close to communicating the rich cosmologies of many Indigenous Peoples. Echoing previous research about Indigenous knowledge (Lambert and Mark‐Shadbolt, 2021), this is a complex entwining of worldviews and motivations, where the line between silencing, co‐opting, misappropriating, and respecting Indigenous knowledge is blurred.

### How do narratives differ from each other?

The narratives and sub‐narratives we identified also had some key differences. The dominant humanitarians act narrative framed Indigenous Peoples (especially Indigenous women) as needing help, justifying a humanitarian presence and aligning with previous work that shows how humanitarians present themselves as alleviating the suffering of helpless civilians (Boltanski, 2000; Franks, 2018). Whether through a humanitarian intervention that provided for basic needs, or through humanitarians ‘empowering’ Indigenous Peoples, the plot fundamentally centres on the importance of humanitarian action. Across most articles under the ‘humanitarians act’ narrative, there were no fictionalised enemies: disasters were predominantly constructed as natural and unpredictable. The exception to this was the ‘humanitarians help Indigenous women help themselves’ sub‐narrative, where Indigenous men were sometimes framed as enemies, especially where the disaster discussed was gendered (such as gender‐based violence).

In stark contrast are the ‘attributing culpability’ narratives, premised on constructing a fictionalised enemy: the State and/or corporations. These entities either create disasters such as statelessness and pollution, or poorly manage disasters such as pandemics. Instead of undertaking direct action, humanitarians witness and report a crisis, responsibilising the international community to act based on violations of human rights. Relative to other narratives, most humanitarians engaging with this one tended only to write in accordance with this narrative and not engage with others, indicating that they likely view themselves as having a specific role in the international humanitarian community: that of witnessing, reporting, and advocating, through legal channels.

The sub‐narrative ‘corporations create disasters’ exclusively focused on the extractive industry in Central and South America. Given that corporations create disasters across other contexts in sectors beyond the extractive industry (Klein, 2007), it is interesting that only these are reported. This could be because the extractive industry has very visible impacts on the environment and health, whereas other industries do not and thus are not used to galvanise action. In doing so, humanitarian communications limit understanding of how corporations create a disaster by masking processes like capitalism. The absence of humanitarian narratives about North America, where Indigenous movements contest extractive industries (Kuokkanen, 2019), is also striking, suggesting that humanitarian crises and disasters are still by and large viewed as something that happen in the distant, different, and separate ‘Global South’ (Atuhura, 2022).

‘The people help the people’ is the only narrative where Indigenous Peoples are not passive, but active: they are the main characters and the only ones who can and will help each other by sharing struggles and standing in solidarity. Like the ‘attributing culpability’ narrative, governments and the international community are complacent at best and discriminatory at worst. Unlike the ‘attributing culpability’ narrative, humanitarians solely witness and report on the situation, but they do not engage in advocacy or lobbying of governments. This narrative is very much focused on grassroots action, which is usually obscured by international humanitarian actors (Lewis, 2019), and follows others (Pearce, 2010; Bebbington, Hickey, and Mitlin, 2013) who underscore that building alliances with grassroots organisations (and seeing themselves as a part of these social movements) is how NGOs should operate.

‘The nation tackles disaster’ is another narrative where humanitarians witness disaster. Unlike the previous ones, which centre primarily on suffering, this narrative is one of a nation triumphing against an external threat. The government is framed as heroic and is helping Indigenous Peoples. While previous narratives allude to the continuation of dealing with a disaster, this narrative reaches an end point where the disaster is over. SIDS such as Kiribati and Samoa are the setting for some of these articles, possibly reflecting views of good governance in these states, which means that the international humanitarian community is more likely to offer support (Weiler, Klöck, and Dornan, 2018).

The final narrative, ‘innovating out of disasters’, also reaches a successful end point: a disaster happens and Indigenous Peoples partner with researchers to combine their knowledge with technology to ‘build back better’. The focus here is on collaboration with researchers, specifically in the form of improving technology. Characters that featured in previous narratives, such as the government, corporations, humanitarians, and the international community, are absent. Instead, the role of technology is emphasised. This was the only narrative that included Indigenous Peoples in North America. Shying away from questions of governance here distracts from political questions about Indigenous–State relations in North America, suggesting technology as the solution. Doing so not only ignores that technology can erode Indigenous Knowledge (Young, 2019), but aligns with previous technocratic views of why disasters happen (such as because of environmental hazards) and how we deal with them (such as through managing the environment). The absence of ‘humanitarians’ contrasted with the presence of technology, and research suggests normative notions of progress in North America relative to other regions reported on. Although the framing of the ‘Global South’ as underprivileged relative to regions such as North America is nothing new (Mohanty, 2003), it is nonetheless striking that Indigenous Peoples in North America are only mentioned under narratives that demonstrate the progress of research and technology.

### Narratives reflect different perspectives about governance

The differences between narratives reflect differences in views on the governance of humanitarian crises and disasters. Broadly, this highlights the power of narratives in communicating the ideal worlds of humanitarians, as well as ideas concerning governance.

Tensions between the ‘humanitarians act’ narrative and the ‘attributing culpability’ narrative align with literature about whether NGOs should work with the system (such as the State) or against it (Ishkanian and Shutes, 2022), with ‘attributing culpability’ narratives working against governments in a ‘naming and shaming’ manner (Hendrix and Wong, 2014). To some degree, this is also reflected in the types of disaster reported too: the ‘attributing culpability’ narrative largely focuses on political disasters, created by the State and/or corporations. Here, the ways in which disasters and crises are not objective categories were especially clear in our results: pollution and police violence were framed as disasters, yet some actors (such as corporations and governments) saw them as opportunities. International humanitarian communications in this regard do not seek to be perceived as neutral, but rather as advocating for Indigenous Peoples in the face of powerful governments and corporations. Conversely, most articles adopting the ‘humanitarians act’ narrative have likely opted to work with the State: they do not explicitly attribute the cause of disaster to be the outcome of the State.

Differing views of governance are also reflected in differences within the ‘humanitarians act’ narrative. In this respect, there is a tension between two sets of sub‐narratives: those that ‘save’ Indigenous Peoples, and those that ‘empower’ them. The ‘saving’ sub‐narratives centred on addressing immediate needs and were relatively short term, whereas the ‘empowering’ sub‐narratives concentrated on tackling inequality, reflecting shifts in viewing disasters as unnatural as well as resilience humanitarianism (Hilhorst, 2018). Although the ‘humanitarians save Indigenous Peoples’ sub‐narrative was the most frequent within the ‘humanitarians act’ narrative, the ‘humanitarians help Indigenous women help themselves’ sub‐narrative was more prominent than saviour narratives about Indigenous women. Such shifts in narrative towards the empowerment of *third world* women and girls is a hallmark of neoliberalism in that it responsibilises individual women and proposes market‐based solutions, distracting from power structures (Roberts and Mir Zulfiqar, 2019; Rosamond and Gregoratti, 2020). Within these narratives women were often framed as activists and protectors of their communities, consistent with Indigenous feminist epistemology (Dulfano, 2015, 2017). Their activism mostly spotlighted patriarchy, gender inequality, and machismo, but these were not contextualised to show how intertwined they are with colonialism (Patil, 2013; Wilson, 2019) or how the gender and gender relations employed are Western constructs that silence others (Momsen, 2002; Medwinter and Rozario, 2021).

What is not mentioned with regard to Indigenous women's activism is resisting neo‐liberalism (such as in the form of extractive industries or liberal feminism). By not doing so, most humanitarian communications do not adequately convey the role of neo‐liberal policies to their audiences, while also silencing elements of Indigenous women's activism (Dulfano, 2017). Instead, communications focus on power structures that may be seen as internal to Indigenous communities. This inhibits audiences from seeing themselves as part of a network implicated in these power relations. Similarly, SIDS were not represented in saviour narratives but were in empowerment narratives, reflecting the presence of empowerment and resilience discourses about these contexts, but not ones around saviourism. This could be because dominant narratives about SIDS have unduly concentrated on vulnerability, which has led to push back by and counter narratives from SIDS' peoples (Kelman, 2018; Teng, 2019). These shifts from ‘saviourism’ to ‘empowerment’ narratives are well documented (see, for example, Hilhorst, 2018), yet there are a surprising number of communications that still use saviour rather than empowerment narratives.

The infrequent emergence of the ‘innovating out of disasters’ narrative aligns with neoliberal ideas about ‘building back better’ (Cheek and Chmutina, 2022), where communities are responsibilised to deal with the risks they face, as well as with techno‐utopianism—the belief that we can innovate our way out of global problems. Both of these concepts have capitalist and/or neoliberal foundations (Sandvik et al., 2014; Bankoff, 2019) that fundamentally ignore questions on inequality. There is a considerable amount of optimism about the possibilities of technologies in the humanitarian sphere, including the use of biometric identification, e‐transfers, and drones (Madianou, 2019), although there are also concerns about surveillance (Lambert and Henry, 2020). Optimism about innovation in the context of our research centred on novel ways to construct buildings by combining Indigenous Knowledge with Western science and technology, rather than the former. This could be due to controversy over the use of some humanitarian technologies, as well as the public–private partnerships involved (see, for example, Madianou, 2019), meaning that although the plotline communicating the importance of innovation and technology is present, it is separated from more controversial debates. This aligns with the growing recognition of Indigenous Knowledge within international DRR policies (Lambert and Scott, 2019). However, as argued by Shaw, Sharma, and Takeuchi (2009) and Lambert and Scott (2019), DRR requires more than scientific and technological advances. Nevertheless, as illustrated by Abdelnour and Saeed (2014) in relation to using stoves to address rape in Darfur, western Sudan, reducing complex issues to ‘manageable problems’ to be solved through technology does not address root causes, even where Indigenous Knowledge has been incorporated.

### The silent influence of audiences

Humanitarian communications do not necessarily reflect the practices of humanitarians. Instead, they are constructed by humanitarians based on ways they believe will bolster their credibility in the eyes of audiences (Gourevitch and Lake, 2011). Narrative analysis, then, reveals values shared by both humanitarian organisations and their audiences. This places some of the above discussion about disasters, gender, and the environment in context.

As alluded to earlier, the very question of what disasters and crises are included/excluded relies upon both humanitarians and audiences agreeing that some process or occurrence can be described as a disaster or crisis, leading to the masking of some events, but we cannot claim that humanitarians do not work on these. Instead, the idea of disaster is co‐constructed by humanitarians and their audiences based upon a shared understanding of a problem, and audiences themselves may not be galvanised to act in these situations, even if they are implicated in them through processes such as imperialism.

Similarly, intersectional feminist approaches to humanitarian response are lacking and needed (Lafrenière, Sweetman, and Thylin, 2019; González Villamizar and Bueno‐Hansen, 2021), yet were mostly invisible in our analysis. Despite calls for intersectional disaster management in some articles (usually around the intersection of ableism and racism), humanitarian communications offered simplified and decontextualised accounts, often staying firmly within the bounds of liberal feminism (such as promoting market‐based solutions) (see also Smith et al., 2022). This was clearest in communications that followed the ‘humanitarians help Indigenous women help themselves’ sub‐narrative. However, the lack of intersectional approaches reflected in our analysis does not necessarily support the argument that the humanitarian organisations included are not concerned with intersectionality. Instead, it may be that humanitarian organisations know that potential donors could be discriminatory based on their own cultures. For instance, it is possible that NGOs do support gender identities that do not conform to Western feminism's gender binary (such as transgender and two‐spirit people), but do not publicise this widely due to rising discrimination of these groups across the donor landscape. As such, the lack of intersectional approaches here could reflect actual humanitarian practice, the donor landscape, or (most likely) both.

### Future research and policy implications

Although ‘the people help the people’ narrative focused on grassroots action, the viewpoints of grassroots groups, volunteers, Global South‐based organisations, and a plethora of other crucial but less visible humanitarians are not reflected here. The actors included are not diverse, possibly reflecting how large, international humanitarian actors often obscure the work and the importance of civil society and volunteers (Lewis, 2019). For example, despite the Wayúu Indigenous group being the focus of several articles, the most representative political organisation of the Wayúu people, the *Sütsüin Jieyuu Wayúu – Fuerza de Mujeres Wayúu* (Wayúu Women Force) (Ulloa, 2020), was not mentioned. Another absence, both in terms of humanitarians and Indigenous Peoples, is evident in regard to the Global East, despite significant humanitarian efforts by these states and the presence of numerous Indigenous groups in this part of the world (Burnasheva, 2019; see also Hadlos, Opdyke, and Hadigheh, 2022). Thus, while the UN and many humanitarians claim to act internationally across a range of contexts, this is clearly not the case (Amin, 2013). Future research should concentrate on the perspectives of other groups to see how narratives compare with those outlined here. Such work should elevate the viewpoints of Indigenous Peoples, such as by examining the impacts of these narratives and how to subvert those that are harmful. Future work could also critically assess other influential actors that are hidden in our work, such as humanitarian action by powerful states in the Global East.

Our research illustrates the oft‐forgotten role of narratives in communicating ideas about governance. Consequently, narratives and narrative analysis have saliency for policy and decision‐making. Broadly, those engaged in policy should be careful about the narratives they construct, as well as about those with which they engage. In the context of localisation, for example, questioning what the end goal of localisation is, and who it benefits, matters. Is localisation being employed to allow powerful actors to evade responsibility? Will it address processes that constitute slow violence and vulnerability? Or is it used by communities themselves as a steppingstone towards self‐determination? More recently, the IPCC's (2022) latest climate change report on impacts, vulnerability, and adaptation draws on Indigenous Knowledge more so than previous iterations. Yet, Indigenous organisations have critiqued the superficial way that this knowledge was incorporated in the process of creating the IPCC (Carmona et al., 2022). When engaging Indigenous Peoples, therefore, it is fundamental that their sovereignty and their rights (both inherent and through the UNDRIP and/or signed treaties) to articulate their own positions are taken seriously. This applies not only to asserting how disasters and crises should be managed, but also to defining and framing what such an event is in the first place. In addition, it is important to engage deeply with their (diverse) perspectives, worldviews, and histories, such as by taking seriously the harm colonialism and its legacies has had on Indigenous communities, and remembering that hegemonic practices (such as humanitarian action, disaster management, and research) are also cultures that are not normal but created. Doing so calls into question the legitimacy of the State and private structures that exert power over Indigenous Peoples through processes such as DRR and humanitarian action (Lambert, 2022). Hence, we call on researchers and practitioners to recognise the colonial and racist roots of disasters, humanitarian crises, and their management and take action that is anti‐racist and moves towards decolonisation^4^ (Fujita, 2020; Lambert, 2022). Thus far, the international humanitarian sector has been resistant to understanding its monopoly, abuse, and misuse of power, which is needed to begin the process of decolonisation (Aloudat and Khan, 2022; Majumdar and Mukerjee, 2022).

Lastly, we encourage organisations producing such communications to explore how care ethics could inform their practice. This would entail emphasising solidarity with people affected by disasters (similar to ‘the people help the people’ narrative), and bringing to the fore the interconnectedness of people, for instance by highlighting a shared history. This would allow audiences to see themselves as connected to those represented through processes in which they are implicated. In terms of reporting in Indigenous contexts, processes of colonisation, colonialism, and imperialism are pertinent. These would need to be contextualised within local settings so that audiences can see how these processes are intertwined with peoples' lives. By facilitating understanding of complex situations and their histories, humanitarian communications would raise consciousness among their audiences about their responsibilities.

## Conclusion

We have examined the narratives these humanitarian communications adopt to convey meaning about disasters and crises in Indigenous contexts. The most invoked narrative focused on the importance of humanitarian action, followed by the one that attributed culpability. A central theme that tied our analysis together concerned how communications conveyed meaning of governance. The ‘humanitarians act’ narrative carved out a role for humanitarians, while remaining either neutral or positive towards government. Conversely, the ‘attributing culpability’ narrative witnessed and reported on disasters created by governments and corporations, calling on the international community to act. The other three less frequent narratives conveyed meaning about the importance of grassroots action (‘the people help the people’), nationalism (‘the nation tackles disaster’), and techno‐utopianism (‘innovating out of disasters’).

Another crosscutting theme was how humanitarian communications mediated the relationship between Indigenous Peoples (as the focus of humanitarian communication) and audiences. While we cannot expect humanitarian communications to represent fully the lived realities of Indigenous Peoples, we can say something about what they choose to include and/or exclude, the ramifications of this, and any possible alternatives. We found that the international humanitarian community's communications limited explicit recognition of the processes that link audiences to Indigenous Peoples. These include processes such as colonialism, neoliberalism, and globalisation. Instead, communications portrayed power structures as internal to communities and/or countries. That these are the ways disasters and crises in Indigenous contexts are represented to many audiences is important because it has the potential to diminish the responsibility of Western audiences to push back against unequal power structures. It raises awareness of Indigenous Peoples lives across the globe, while inhibiting crucial questions of why they experience disasters and crises the way they do (and indeed, so differently to settlers) (Lambert, 2022).

It is well documented within the humanitarian literature that this is how humanitarian communications portray suffering. This does not mean that we should become complacent about this, though. Instead, it is imperative to think of alternative ways to communicate meaning and, where appropriate, galvanise a response. Here we draw on care ethics, which in a humanitarian context moves away from viewing people as ‘distant sufferers’ and towards people connected with each other through complicated histories. In doing so, audiences gain a more complete understanding of how they themselves are implicated in oppressive processes and can take action to address this fact.

## Acknowledgements

We would like to thank formally three anonymous peer reviewers whose comments undoubtedly strengthened the quality of this manuscript. We would also like to extend our gratitude to Melanie Flynn for her insights and expertise, as well as her support throughout the process of this research.

Anuszka Mosurska was funded by the Economic and Social Research Council (ESRC)'s White Rose Doctoral Training Partnership (project number: 2113218). Aaron Clark‐Ginsberg was funded by the National Science Foundation/National Oceanic and Atmospheric Administration, under the project titled ‘Belmont Forum Collaborative Research: Community Collective Action to Respond to Climate Change Influencing the Environment‐health Nexus’ (award number: 2028065). Susannah M. Sallu was funded by the ESRC Centre for Climate Change Economics and Policy – Transition Phase (award number: ES/R009708/1). Katy Davis was funded by a Priestley Scholarship from the University of Leeds.

## Data availability statement

The data that support the findings of this study are available from the corresponding author upon reasonable request.

## Endnotes

2 We have sought to be specific in our referencing here by including the last name of those reporting on behalf of humanitarian groups (for instance, Benrey, Jahan, and Macheka). However, many humanitarian organisations do not attribute their communications to a specific person. Where this is the case, we have just included the organisation (such as European Union (EU), Médecins Sans Frontières (MSF), and Salesians).

3 We recognise that there is debate regarding defining disasters, and some may not view armed conflict as a disaster. As we did not limit our search by categories of disaster or crisis, it would not have made sense to exclude communications because they were about conflict. Although we acknowledge that armed conflict includes its own specificities (see, for example, Mena and Hilhorst, 2022), we follow numerous disaster researchers (see, for example, Peters and Kelman, 2020; Hewitt, 2021) in arguing for greater integration of conflict into disaster research.

4 Following Tuck and Yang (2012), we recognise that decolonisation will look very different across contexts due to the diversity of Indigenous Peoples and the complex histories and processes of colonisation imposed on them. For this statement to have meaning, researchers and practitioners wishing to do this would need first to attend to these histories and then understand how they are implicated within them and how to push back appropriately.
